# Clinical and Ultrastructural Studies of Gelatinous Drop-Like Corneal Dystrophy (GDLD) of a Patient with *TACSTD*2 Gene Mutation

**DOI:** 10.1155/2019/5069765

**Published:** 2019-08-20

**Authors:** Ali Masmali, Aljoharah Alkanaan, Hind M. Alkatan, Omar Kirat, Abdullah Ayidh Almutairi, Turki Almubrad, Saeed Akhtar

**Affiliations:** ^1^Cornea Research Chair, Department of Optometry, College of Applied Medical Sciences, King Saud University, Riyadh, Saudi Arabia; ^2^King Khalid Eye Specialist Hospital, Riyadh, Saudi Arabia

## Abstract

**Purpose:**

To describe clinical, molecular genetics, histopathologic and ultrastructural findings of gelatinous drop-like corneal dystrophy (GDLD) (OMIM #204870) in a Sudanese patient.

**Method:**

An ocular examination revealed the onset of GDLD in a Sudanese patient (50 years old) at King Khalid Specialist Hospital, Riyadh. The 333 sequence variants in 13 GDLD genes of a DNA sample were screened by Asper Ophthalmics Ltd. It was further confirmed by sequencing. The patient had undergone a penetrating keratoplasty in the right eye. The corneal tissue was processed for histopathology and ultrastructural studies.

**Results:**

Slit-lamp observation showed grayish-white multiple superficial corneal nodules of various sizes in the left and right eye. Both corneas became clear after the surgery. The GDLD deposits in the subepithelial region and in the anterior stroma were confirmed by PAS staining and their apple-green birefringence under polarized light. Ultrastructurally, the amyloid fibrils were very thin and grouped in whorl-like structures, which caused splits between and within the stromal lamellae. Collagen fibrils (CFs) and keratocytes had degenerated. A homozygous c.355T > A mutation in exon 1 of the *TACSTD*2 (*M*1*S*1) gene was detected, and alteration of the amino acid (p.Cysl19Ser in NCBI entry NP_002344.2) was observed.

**Conclusion:**

In our patient with GDLD, a “c.355T > A” mutation in exon 1 of *TACSTD*2 was detected and believed to be responsible for the alteration of the amino acid leading to the formation of the amyloid deposits. The deposits caused the ultrastructural degeneration of epithelium, Bowman's layer, stroma, and keratocytes of the GDLD cornea.

## 1. Introduction

Gelatinous drop-like corneal dystrophy (GDLD; OMIM #204870) is characterized by the presence of multiple, gelatinous-like deposits in the subepithelial and stromal region of the cornea. The formation of the gelatinous lesions is due to deposition of amyloid in the superficial cornea [1, 2]. The deposits spread within the stroma leading to the impairment of vision. GDLD is an inherited ocular disease, which manifests commonly within the first-second decade of life (8 to 18 years). Nakaizaumi [1] was the first to describe it as gelatinous drop-like dystrophy, in Japan.

GDLD is caused by a mutation in the tumor-associated calcium signal transductor 2 (*TACTD*2) gene which is located on the short-arm of chromosome 1 [3, 4]. The disease is relatively common in Japan (1 in 333,000) but also occurs in other parts of the world such as India, China, and Europe [3–8]. Markoff et al. [8] reported the occurrence of GDLD in one Turkish family. The authors investigated bilateral GDLD corneal amyloidosis in two teenage girls (8 and 13 year) and showed a novel *TACSTD*2 mutation, c.653delA, occurred in both patients. Alavi et al. [9] have investigated mutations in 13 Iranian families with GDLD and found an association with four mutations in *TACSTD*2 (C66X, F114C, L186, and E227) with GDLD. The mutations C66X, F114C, and L186 were novel, whereas E227K was common among the members of the 10 families.

There are few studies carried out on the ultrastructure of the lamellae, collagen fibrils, and proteoglycans of the GDLD cornea. It has been noted that, in the early stage of GDLD, the basal lamina and Bowman's layer (BW) are intact, but in the later stages, basal epithelial cells and basal lamina degenerate [10]. Kinoshita et al. [11] reported the presence of amyloid deposits in between the collagen fibrils of the stroma causing degeneration and disorganization of stromal collagen fibrils (CFs). Büchi et al. [12] reported that the amyloid deposits were mostly rounded and contained irregularly running amyloid fibrils of various thicknesses ranging from nanometers to the several microns. There was a large amount of lactoferrin and keratoepithelin (βig-h3) observed in the ultrastructural nodular deposits in the cornea of a child diagnosed with GDLD [13].

In the present study, we investigated the genetic, histopathological, and ultrastructure features of a Sudanese patient affected by GDLD. The patient was diagnosed at King Khalid Specialist Eye Hospital, Riyadh (to our knowledge, this is the first case of GDLD diagnosed in Saudi Arabia).

## 2. Methods

### 2.1. Ethical Approval

The Local Ethical Committee King Saud University, Saudi Arabia, ethically approved the use of tissue procurement. All experiments were done in accordance with the guidelines of “Standing Committee for Research Ethics on Living Creatures (SCRELC)” Saudi Arabia. The policy is available at https://www.uod.edu.sa/sites/default/files/resources/implementing_regulations_0.pdf.

### 2.2. Clinical Details (Figures 1(a)–1(d))

A 50-year-old Sudanese patient was referred to King Khalid Specialist Hospital, Riyadh. The patient had been living alone in Riyadh, Saudi Arabia, since 1998. He complained of the sensation of having a foreign body in his eye and reported the gradual decline of vision in both his eyes which developed over many years. He was diagnosed with “typical mulberry-type” GDLD. There was no obvious history of similar ocular conditions in his family.

In August 1997, the patient had penetrating keratoplasty (PKP) in both the right (OD) and left eye (OS). Recurrent amyloid occurred on the PKP cornea. In March 1999, the superficial keratectomy was carried out to the OD, and in January 2005, lamellar keratoplasty (LKP) was carried out to the OS (left eye). The visual acuity in the right eye (OD) was 20/400 and in the left eye (OS) was 20/100 after the last surgery. The intraocular pressure was 18 mmHg in the right eye and 12 mmHg in the left eye.

**Figure 1:**
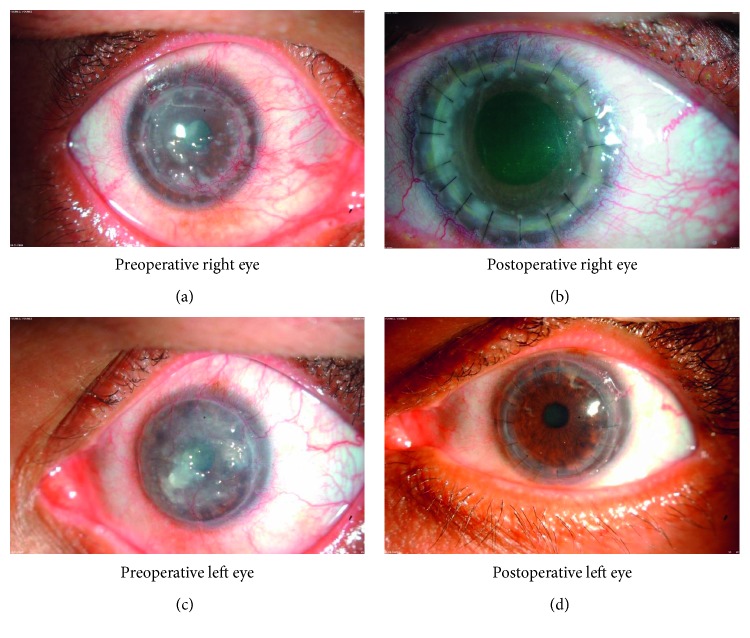
Clinical photograph of preoperative and postoperative appearance of both eyes of the GDLD cornea of the patient. (a) Slit-lamp photograph of the right cornea showing grayish-white multiple superficial corneal nodules of various sizes; (b) slit-lamp photograph of right eye showing clear cornea after surgery; (c) slit-lamp photograph of the left cornea showing dense grayish-white multiple superficial corneal nodules of various sizes; (d) slit-lamp photograph of left eye showing clear cornea after surgery.

In May 2011, both eyes presented with multiple superficial corneal nodules of various sizes, the whole cornea was involved including the graft (Figures 1(a) and 1(b)). The left (OS) lamellar corneal graft was still clear. He had mild cataract in both eyes.

In May 2011, the patient had another penetrating keratoplasty to the right eye (OD). Tissue was removed by penetrating keratoplasty of the right eye and was processed for light and electron microscopy. His vision improved without correction. The visual acuity (VA) was OD 20/40 and VA OS 20/50 (Figures 1(c) and 1(d)).

### 2.3. Genetic Analysis

A blood sample from the patient was sent to Asper Ophthalmics Ltd. for screening against 333 known sequence variants in 13 genes in the corneal dystrophy panel (*COL*8*A*, *TGFBI*, *VSX*1, *CHST*6, *KRT*3, *KRT*12, *GSN*, *TACSTD*2, *CYP4V*2, *SOD*1, *ZEB*1, *SLC*4*A*11, and *UBIAD*1). The mutation in *TACSTD*2 was further confirmed by sequencing, carried out by Asper Ophthalmics Ltd.

### 2.4. Light Microscopy and Electron Microscopy

Half of the cornea was fixed in 10% formalin overnight and then washed with phosphate buffer, followed by dehydration in ethanol (70% to 100%). The tissue was embedded in paraffin. Ten micron sections were cut and stained with the standard method of hematoxylin and eosin (H&E). The presence of GDLD was confirmed by classical staining for amyloid with PAS, Congo red, and polarizing Congo red.

For electron microscopy, half of the cornea was fixed in 2.5% glutaraldehyde containing 0.05% Cuprolinic blue in sodium acetate and magnesium chloride. The tissue was dehydrated in ethanol (70% to 100%) and embedded in Spurr resin at 70°C for 8 hrs [13]. Ultrathin sections were cut with RMC ultracut microtome and stained with uranyl acetate and lead citrate. The sections were observed under transmission electron microscope JOEL 1400. The digital images were taken with a bottom mounted Quamesa CCD camera.

## 3. Results

### 3.1. Genetic Analysis of GDLD Cornea

Our genetic analysis has shown that a homozygous c.355T > A mutation (rs80358227) in exon 1 of the *TACSTD*2 (*M*1*S*1) gene was present in our GDLA patient. As a result of this mutation, a pathogenic amino acid alteration (p.Cysl19Ser in NCBI entry NP_002344.2) had occurred (Table 1). This mutation has been reported previously in two families of Tunisian origin [14].

### 3.2. Histopathological Observation of GDLD Cornea

**Figure 2:**
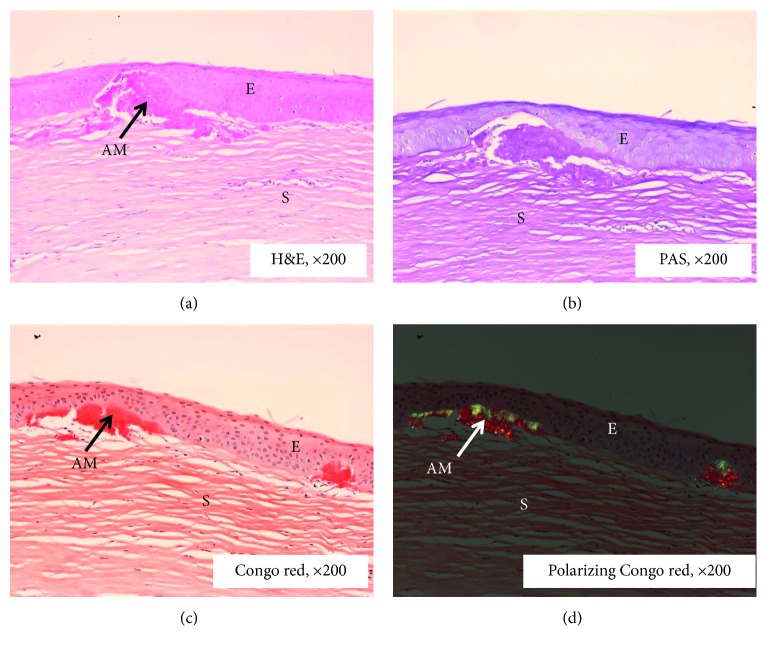
Histopathological analysis of GDLD cornea. (a) The degenerate epithelium, amyloid deposit, and absence of Bowman's layer and stroma (H and E). Notice the absence of Bowman's layer. (b) Part of the cornea showing detached epithelium, stroma, and amyloid deposits positively stained with PAS; (c) part of the cornea showing detached epithelium, stroma, and amyloid deposits positively stained with Congo red; (d) part of the cornea showing amyloid deposits with polarizing Congo red. AM: amyloid deposit; E: epithelium; S: stroma.

In the GDLD cornea, the epithelium was very thick and had degenerated (Figures 2(a) and 2(b)). The aggregates of amyloid fibrils were present below the epithelial region and in various parts of the stroma. In some parts, the epithelium was elevated and thin due to the accumulation of the amyloid deposits (Figures 2(a) and 2(b)). Keratocytes had also degenerated. The deposits were Congo red- and PAS-positive and showed apple-green birefringence under polarized light (Figures 2(b)–2(d)).

### 3.3. Electron Microscopy of GDLD Cornea

**Figure 3:**
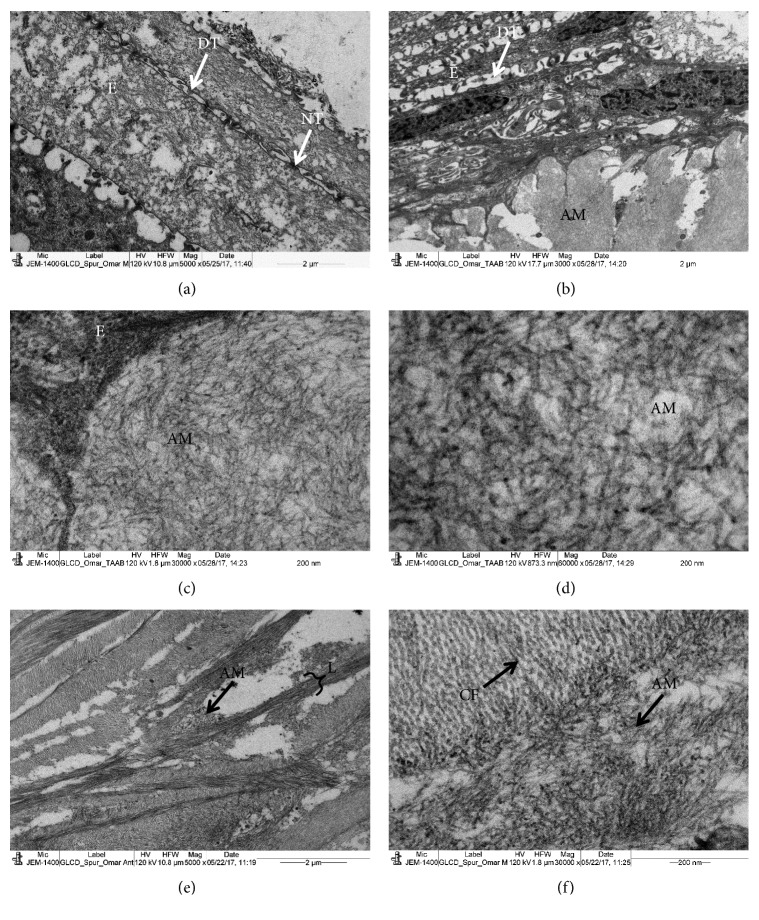
Electron micrograph of GDLD cornea. (a) Part of a degenerated epithelium containing degenerated tight junctions and normal tight junctions. Note that most of the tight junctions are degenerated forming electron-lucent spaces. (b) Numerous running randomly amyloid fibrils deposits at subepithelial region; (c, d) a network of amyloid fibrils at high magnification at subepithelial region; (e) part of the anterior stroma showing a degenerated lamellae and electron-lucent spaces; (f) the stromal amyloid fibrils associated with collagen fibrils. AM: amyloid deposits; CF: collagen fibrils; E: epithelium; L: lamella, TD: degenerated tight junctions; NT: normal tight junctions.

The epithelium contained numerous electron-dense vacuoles and apoptotic nuclei (Figures 3(a) and 3(b)). Most of the tight junctions had degenerated and appeared as electron-lucent vacuoles. There were some normal tight junctions with electron-dense material around them (Figures 3(a) and 3(b)). A thick band of amyloid deposits were present in the subepithelial region (Figure 3(b)), and these deposits consisted of very thin amyloid microfibrils running randomly (Figures 3(c) and 3(d)). Most parts of the epithelium had detached from the basement membrane and BW due to subepithelial growth of the amyloid deposits. In some places the amyloid microfibrils were closely attached to the surface of Bowman's layer (Figure 3(c)). Bowman's layer was thick and contained very long collagen fibrils and large deposits of amyloid fibrils (Figure 3(c)). These amyloid fibrils were densely merged with each other (Figure 3(d)).

**Figure 4:**
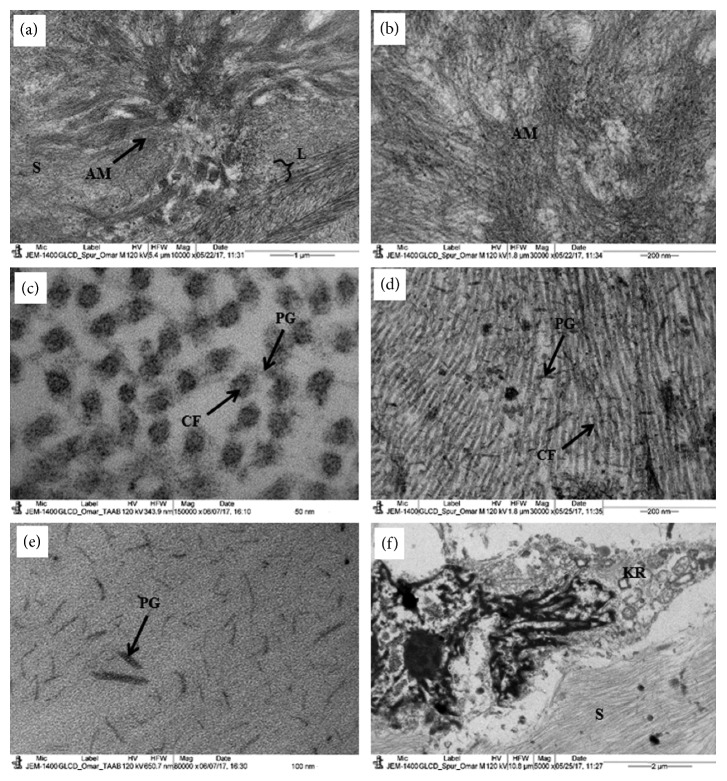
Electron micrograph of the GDLD cornea. (a) A whorl-like structure of the amyloid deposit containing very thin amyloid fibrils. (b) The electron-dense and electron-lucent amyloid fibril in the deposit. (c) Part of the stroma showing cross section of randomly distributed collagen fibrils; (d) part of the stroma showing randomly longitudinally running collagen fibrils with large proteoglycans; (e) part of the stroma showing very large proteoglycans (no staining of Ua and Pb); (f) part of the stroma showing a degenerated keratocyte containing large apoptotic nucleus. AM: amyloid deposits; CF: collagen fibrils; KR: keratocyte L: lamella; PG: proteoglycans; S: stroma.

The deposits of the amyloid fibrils were also observed in between the lamellae of the anterior stroma creating large lucent spaces and vacuoles (Figures 3(e) and 3(f)). The amyloid fibrils were also present within the lamella replacing the collagen fibrils and creating electron-lucent vacuoles (Figures 3(e) and 3(f)). In some parts of the stroma, the deposits of amyloid fibrils were whorl-like structures showing the emergence of the amyloid fibrils from the center of the whorl (Figures 4(a) and 4(b)). These whorl-like structures had electron-dense and electron-lucent areas (Figures 4(a) and 4(b)). The uniform distribution of the collagen fibrils had been disturbed, and collagen fibrils were of variable diameters (Figure 4(c)). The proteoglycans were very large and randomly distributed in between the longitudinally running collagen fibrils (Figures 4(d) and 4(e)). The keratocytes had degenerated and surrounded the amyloid fibrils (Figure 4(f)).

## 4. Discussion

Our present studies revealed that the gelatinous drop-like deposits in the cornea of a Sudanese patient (50-year-old) living in Saudi Arabia was caused by the occurrence of a homozygous c.355T > A mutation in exon 1 of the *TACSTD*2 (*M*1*S*1) gene. It has been predicted that the mutation in the gene causes pathogenic amino acid changes (Cl19S) in the thyroglobulin-like repeat region. Ren et al. [14] identified the same c.335T > A mutation in two families of Tunisian origin.

In our patient, the epithelium was full of electron-lucent spaces instead of tight junctions. We believed that the degenerated tight junctions coalesced to each other forming electron-lucent spaces (Figures 3(a) and 3(b)). The deposits in our patient were present below the epithelium and in the anterior stroma and showed apple-green birefringence under polarized light. These deposits were positive to Congo red and PAS staining. Ultrastructural studies showed that these deposits had a whorl-like structure in the stroma containing very thin electron-dense and electron-lucent amyloid fibrils. The lamellae of the stroma were spars and degenerated due to the presence of the deposits between the lamellae. The deposits were also present within the lamellae causing degeneration of the collagen fibrils and proteoglycans.

Nakaizumi [1] for the first time reported that GDLD is an autosomal recessive disease and occurs rarely. The important characteristic feature of the disease is the aggregation of the amyloid deposits at the subepithelial region of the cornea bilaterally. There are several other diseases such as Avellino corneal dystrophy (ACD) and lattice corneal dystrophy (LCD) which are caused by the aggregation of the amyloid fibrils. GDLD has been classified into 4 types: (1) band keratopathy, (2) stromal opacity, (3) kumquat-like, and (4) typical mulberry [2, 15]. According to the classification, our patient had “typical mulberry-type” GDLD. It has been reported that the clinical symptoms of GDLD appear as nodular deposits in the central part of the cornea in the first decade of life. In the later stages of life, these deposits increase in number and coalesce to produce a whitish-yellow mulberry appearance [9, 10].

The presence of amyloid deposits below the epithelial region and anterior stroma of a GDLD cornea have been reported by previous authors [3, 9]. Akhtar et al. [13] reported that the epithelium contained apoptotic nucleus and the BW was replaced by pannus connective tissue. The authors reported that the cause of the disease was the mutation in gene M1S1, in which substitution of aspartic acid to alanine had occurred [13]. In our patient, most parts of the epithelium were destroyed due to the development of amyloid deposits and the thickening of the BW. Similar to these previous observations [13], we also found the stromal collagen fibrils were sparse, degenerated, and disorganized, and the proteoglycans were large in between them. Very fine amyloid microfibrils were observed throughout the stroma and BW.

Tsujikawa et al. [16] suggested that the GDLD in Japanese families is caused by the mutation in the *M*1*S*1 (alias *TROP*-2) gene. Calabrese et al. [17] revised the location of the *M*1*S*1 in the gene nomenclature and renamed it as a tumor-associated calcium signal transducer 2 (*TACSTD*2) gene. The mutations in the tumor-associated calcium signal transducer 2 (*TACSTD*2) gene are located on chromosome 1 (ip32) which leads to the development of the GDLD disorder [3, 16]. In patients suffering with GDLD, 24 mutations have occurred in the *TACSTD*2 *gene* [3, 9, 18, 19]. The genetic heterogeneity of the GDLD was also reported when three mutations in the *TACSTD*2 were not found [6, 9, 13, 14]. A founder effect has been reported for GDLD-causing mutations in Iran and Japan identified Q118X mutations in *TACSTD*2 in 4 separate families from Japan [9, 16]. Investigation of additional genetic markers in the region confirmed a founder effect, despite extensive phenotypic variability that included family members who suffered from atypical amyloidosis not initially diagnosed as GDLD. Also, as mentioned above, in a study of 13 unrelated families from Iran diagnosed with GDLD, Alavi et al. [9] detected an E227K mutation in *TACSTD*2 in 10 patients, also finding evidence of a founder effect for this variant.

The *TACSTD*2 gene product is a multimodule transmembrane glycoprotein of 323 amino acids. It is 2.07 kB in length and has one exon encoding the tumor-associated antigen. This protein is a monomeric cell surface glycoprotein expressed in the cornea, trophoblast, and most carcinomas [3, 20]. The *TACSTD*2 protein plays an important role as an adhesion receptor between cancer cells and as a calcium signal transducer [9, 18, 21]. It is believed that, in the GDLD cornea, the abnormal secretion of the *TACSTD*2 protein causes a high permeability or perforation in the epithelium of GDLD that leads to the formation of amyloid deposits and pathogenicity of the disease [9, 11, 22].

Recently, Nakatukasa et al. [23] reported the direct binding of the *TACSTD*2 protein to CLDN1 (claudin protein) and CLDN7 proteins which protects them from degradation by the ubiquitin-proteasome system [24]. Claudin plays an important role in the formation of tight junctions between the epithelial cells through calcium-independent cell-adhesion activity. The tight junctions define the border between the apical and basolateral compartments of the epithelial cells and control the lateral diffusion of the lipids and proteins between the cells [25].

In our patient, a homozygous c.355T > A mutation in exon 1 of the *TACSTD*2 (*M*1*S*1) gene caused a lack of functional *TACSTD*2 protein. Perhaps due to the absence of the *TACSTD*2 protein, loss of tight junction integrity may have caused the formation of large vacuoles which led to the increased permeability of the corneal epithelium, ultimately leading to the subepithelial and stromal deposition of amyloid in the cornea. These amyloid deposits caused degeneration of the collagen fibrils and proteoglycans leading to corneal opacity.

## Figures and Tables

**Table 1:** Genetic analysis of GLCD. DNA sequence variation that occurs in at least 1% population and is nonpathogenic; DNA sequence variation that is pathogenic or possibly pathogenic.

Sample ID	Gene	Exon	Nucleotide change	Amino acid change	Genotype results	Zygosity	Type of variation	Verified sequence
Male	*TACSTD*2	Ex1	c.355T > A	p.Cys119Ser	A/T	HOM	Mut	+

SNP: single nucleotide polymorphism, HOM: homozygous, HET: heterozygous, and Mut: mutation.

## Data Availability

The data used to support the findings of this study are included within the article.
